# A Study of Hot Deformation Behavior of T15MN High-Speed Steel during Thermal Compression

**DOI:** 10.3390/ma15093017

**Published:** 2022-04-21

**Authors:** Bo Zhao, Zhipei Chen, Changchun Ge

**Affiliations:** School of Materials Science and Engineering, University of Science and Technology Beijing (USTB), Beijing 100083, China; chen_zp0305@sina.com

**Keywords:** high-speed steel, thermal deformation, cellular automaton method, dynamic recrystallization, microstructural evolution

## Abstract

The hot deformation behavior of T15MN high-speed steel during thermal compression was studied by experiment and simulation. Specifically, the hot compression test was carried out on a Gleeble-1500 thermal-mechanical simulator at temperatures from 1273 to1423 K and strain rates from 0.01 to 10 s^−1^ with the deformation degree of 60%. It was found that all the flow stress curves were characterized by a single peak, indicating the occurrence of dynamic recrystallization (DRX), and flow stress will increase with increasing strain rate and decreasing deformation temperature. Based on the experimental data, the constitutive equations and thermal activation energy were obtained ($Q_{\mathrm{act}}$ = 498,520 J/mol). Meanwhile, a cellular automaton model was established via the MATLAB platform to simulate the dynamic recrystallization phenomenon during hot deformation. The simulation results indicate that a good visualization effect of the microstructural evolution is achieved. Both increasing deformation temperature and decreasing strain rate can promote the increase in the average size and volume fraction of recrystallized grains (*R*-grains). Additionally, the calculated flow stress values fit in well with the experimental ones in general, which indicates that the established CA model has a certain ability to predict the deformation behavior of metal materials at elevated temperatures.

## 1. Introduction

High-speed steel (HSS) is widely used for cutting tools due to the advantages of high hardness, high wear resistance, good toughness, and particularly excellent elevated-temperature properties (<600 °C) [1,2,3]. T15MN steel with an alloy content of more than 28% belongs to a kind of high-strength, high- hardness HSS, and is mainly used for fabricating cutting tools for processing high-strength steel, heat-resistant steel, superalloys, or other difficult-to-machine materials. In recent years, high-alloy HSS obtained by the cast and wrought processing route has attracted great attention in the fields of molds, rolls, and bearings owing to its excellent high-temperature resistance and wear resistance, which can not only improve the service life of relevant components, but also reduce the manufacturing cost of the components and parts. From an interrelationship viewpoint, the final mechanical properties of the material are often largely determined by its microstructural morphology [4]. For as-cast HSS, thermo-mechanical processing (TMP), such as the use of hot rolling or hot forging technology, is generally a necessary step for eliminating microstructure defects such as the network eutectic structure for improving the comprehensive performance of materials. Therefore, an in-depth study on microstructural evolution during TMP is of great help for optimizing the mechanical properties of T15MN steel. However, the actual TMP is very complicated, especially for high-carbon and high-alloy materials, due to their complex alloy systems and special microstructures. The hot deformation process of metals or alloys is accompanied by the evolution of their microstructure and properties, which are determined by the combined action of multi-physical mechanisms, such as work hardening, dynamic recovery (DRV), dynamic recrystallization (DRX), and static recrystallization (SRX) [5]. Among these mechanisms, DRX is one of the most important microstructural events occurring in thermo-mechanical processing, and has the function of restoring and homogenizing the deformed matrix, refining the grain, eliminating defects in the microstructure, reducing the stress level, and so forth [6,7]. An understanding of DRX behavior is thus of great significance for adjusting the grain structure and improving the mechanical properties of the material [8].

So far, the conventional means of performing experimental research on the microstructures of materials at different scales has mainly consisted of the use of optical microscope (OM), scanning electron microscopy (SEM), electron backscattered electron diffraction (EBSD), transmission electron microscopy (TEM), etc. These detection methods provide great assistance in studying the microstructure characteristics of materials, whereas microstructural evolution during DRX is discontinuous, which is difficult to track during experiments. Fortunately, the multiscale simulation of the microstructure and properties of materials has gradually become more mature due to the continuous development of computer technology. The common simulation methods for predicting the microstructure and properties of materials consist of finite element (FE), Monte Carlo (MC), phase field (PF), cellular automata (CA). Among these, FM method has advantages in the simulation of macroscopic physical fields such as flow field, temperature field, and electromagnetic field. While the others are regularly used for studying the microstructural evolution of materials on the mesoscopic scale. Compared with the PF method and the MC method, which have more complicated models, substantial computation, and slower calculation speeds, the CA method possesses the unique advantage of building spatial topological relations evolving over time, and can simulate the spatiotemporal evolution of complex systems more simply and efficiently [9]. Because of a more adequate consideration of the physical mechanism of transformation, it has therefore also been widely demonstrated in recent years that the CA method can provide a visible analysis of the virtual microstructural evolution of metal materials well, especially the DRX phenomenon. The metals and alloys involved are mainly composed of general steels [10], Ni-based superalloys [11], magnesium alloys [12], austenitic stainless steel [13], titanium alloy [14], aluminum alloy [15], etc.

For HSS materials, a handful of studies on hot deformation behavior have also been carried out. For example, Ardakani et al. [16] studied the effects of temperature and strain rate on the hot deformation behavior of AISI H10 tool steel. Qu et al. [17] researched the influence of mischmetal on the hot deformation behavior of as-cast AISI M2 high-speed steel. Liu et al. [18] investigated the hot deformation behavior of the 1.15C-4.00Cr-3.00V-6.00W-5.00Mo powder metallurgy high-speed steel. Lin et al. [19] studied the hot deformation behavior and processing map of spray formed M3: 2 high-speed steel. It was found that a uniform and fine *R*-grain structure could be obtained under suitable hot working conditions. However, little information about the computational simulation of DRX for high-speed steel materials is available, and there is a lack of relevant literature on predicting its high-temperature deformability and microstructural evolution. To optimize TMP, especially for steel grades with a wide variety of elements and high alloy content, it seems to be an extremely rewarding attempt to develop an efficient computing model of DRX behavior.

In this study, a series of hot compression tests were performed on the T15MN HSS in the temperature range of 1273 K to 1423 K and the strain rate range of 0.01 s^−1^ to 10 s^−1^. Based on the stress–strain curves, the material constants of the alloy steel are determined through a reverse analysis method, and then the constitutive equations and thermal activation energy of the material can be obtained. Additionally, a two-dimensional DRX-CA model is established for a visual simulation of the microstructural evolution during hot deformation. The effect of thermal deformation parameters (including strain rate, deformation temperature and deformation degree) on the DRX behaviors during hot deformation are comprehensively analyzed. The results of the numerical simulation are well consistent with the experimental ones, in general. The main purpose of this research is to provide a certain reference for the prediction and regulation of microstructure.

## 2. Materials and Methods

**Figure 1:**
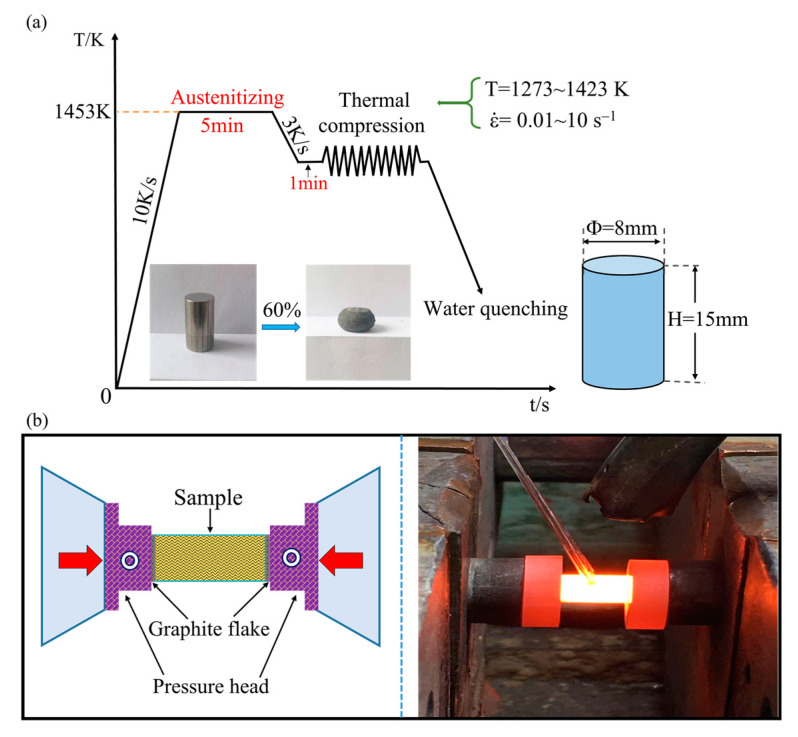
(**a**) Schematic of thermal compression conditions and (**b**) deformation testing.

**Figure 2:**
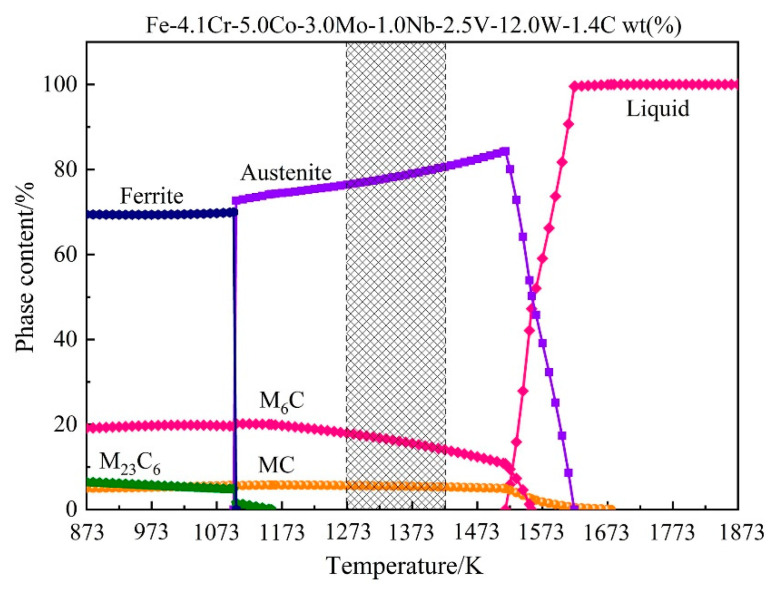
Phase equilibrium diagram of T15MN HSS at 873–1873 K.

The studied material in this experiment is one of the high-speed steel materials, and its specific chemical composition is given in Table 1. The experimental steel was smelted in a 5 kg vacuum induction furnace and then cast into an ingot. The cylindrical samples with a height of 15 mm and a diameter of 8 mm were machined from the as-received ingot at about one-third from the top. Before the thermal deformation, the surfaces of all samples were mechanically polished and then cleaned ultrasonically in alcohol. The isothermal hot compression testing was performed on a Gleeble-1500 thermo-simulation machine with conventional grips. The strain rate ranged from 0.01 s^−1^ to 10 s^−1^ and the deformation temperature ranged from 1273 K to 1423 K. The compression deformation degree of the samples was 60% of the initial height, approximately corresponding to the true strain of 0.9. Figure 1 displays the schematic diagram of hot compression experiments. First, each sample was heated to 1453 K at a rate of 10 K/s and soaked for 5 min for austenitizing to obtain a uniform distribution of the alloying elements before testing. Subsequently, the samples were cooled to the designed deformation temperatures (1273 K, 1323 K, 1373 K and 1423 K) at a speed of 3 K/s, and isothermally maintained for 1 min to reduce the temperature gradient. Finally, the samples were compressed to the extent of 60% deformation by single-pass isothermal compression at various strain rates of 0.01 s^−1^, 0.1 s^−1^, 1 s^−1^ and 10 s^−1^. Upon completion of compression, water quenching was adopted immediately to retain the high-temperature deformed morphology of samples. The deformed samples were cut along the compression axis, mechanically polished, and chemically etched for the observation of deformed microstructure (1/3 along the edge of the longitudinal section) by a Leica optical microscope (OM). The thin graphite and tantalum were placed between the machine head and samples to reduce the friction between them during compression and ensure the samples undergo a relatively uniform deformation. After completing the compression experiment, the flow stress values were automatically stored as a function of each deformation temperature and strain rate. Additionally, the equilibrium phase diagram of T15MN HSS was also calculated using thermodynamic software JMatPro, as shown in Figure 2. The pink shaded area in Figure 2 is the temperature range selected for the hot compression test. Before deformation, the microstructure of the matrix is mainly composed of austenite phase and part of M_6_C-type and MC-type carbides.

## 3. Modeling of DRX Behavior by CA Method

### 3.1. Introduction to the CA Model

**Figure 3:**
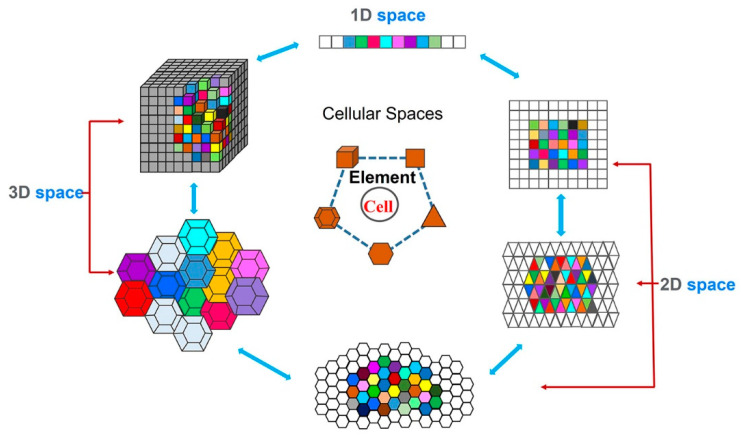
The elements of the cell and the spatial dimensions of the simulation.

The CA method is a grid mathematical model that is discrete in time, space, and state [20]. By pre-setting certain local or global transformation rules, the state of a cell is changed to effectively simulate the spatiotemporal evolution process of some complex physical reaction systems in one-dimensional, two-dimensional, or three-dimensional space. One CA model is mainly composed of a component cell, cell lattice, neighborhood type and transformation rules [21]. Figure 3 reveals the elements of the cell and the spatial dimensions of the simulation. The elements of cell are mostly triangle, square, regular hexagon, regular tetrahedron, regular hexahedron, etc. The neighboring types of Von Neumann and Moore are typically used in two-dimensional space, and consider the nearest and second-nearest neighbors. Relatively speaking, the cellular transformation rule is the core of the entire CA model, because it can be viewed as a dynamic function to determine the dynamic evolution law of the cell state. Therefore, it is a critical link in establishing the appropriateness of the evolution rules with the characteristics of random probability, which directly affects the accuracy of the simulation results. For this purpose, the specific physical reaction process needs to be analyzed to identify its internal connection. The state value of each cell in the model at time t + 1 is determined by its own state variables and the state variables of its adjacent cells at time t by following a deterministic or probabilistic transformation rule [22]. For example, two common types of evolution are as follows [23].

For Von Neumann neighborhood type:

(1) $$\xi_{i,j}^{t+\Delta t}=f\left(\xi_{i-1,j}^{t},\;\xi_{i,j-1}^{t},\;\xi_{i,j}^{t},\;\xi_{i+1,j}^{t},\;\xi_{i,j+1}^{t}\right)$$

For Moore neighborhood type:

(2) $$\xi_{i,j}^{t+\Delta t}=f\left(\xi_{i-1,j-1}^{t},\;\xi_{i,j-1}^{t},\;\xi_{i+1,j-1}^{t},\;\xi_{i-1,j}^{t},\;\xi_{i,j}^{t},\;\xi_{i+1,j}^{t},\;\xi_{i-1,j-1}^{t},\;\xi_{i,j+1}^{t}\;,\xi_{i+1,j+1}^{t}\right)$$

where $\xi_{i,j}^{t+\Delta t}$ represents the state value of the cell site (i, j) at time t + Δt; The function *f* denotes the transformation rules of the cellular automata.

### 3.2. Model of Dislocation Density Evolution

During hot compression of the samples, the evolution of dislocation density inside grains is controlled by the combined action of work hardening and dynamic softening. The softening mechanism consists mainly of DRV and DRX. The Kocks-Mecking (K-M) model is dedicated to describing the variation of dislocation density of cell siting (i, j) in a two-dimensional plane, which is a function of strain and can be expressed as [24]:

(3) $$d\rho /d\varepsilon =k_{1}\sqrt{\rho }-k_{2}\rho$$

where $k_{1}$ and $k_{2}$ represent the hardening parameter and softening parameter respectively, both of which can be calculated by Equations (4) and (5):

(4) $$k_{1}=2\theta_{0}/\alpha \mathrm{Gb}$$

(5) $$k_{2}=2\theta_{0}{/\sigma }_{s}$$

Here, $\theta_{0}$ is the work hardening rate, which can be obtained from the first derivative of true stress–strain curves in the work hardening stage; h is the interaction coefficient of dislocation density, generally taken as 0.5 for most metals; $\sigma_{s}$ is the steady-state stress (MPa); b is the Burgers vector; G is the shear modulus varying with temperature and obtained by linear regression analysis, given by:

(6) $$G=G_{0}[1-\frac{T-300}{T_{m}-293}]$$

where $G_{0}$ is the shear modulus at room temperature; T and $T_{m}$ represent the deformation temperature and the melting point corresponding to the material, respectively. The relationship between the flow stress and average dislocation density can be expressed as:

(7) $$\sigma =\mathrm{hGb}\sqrt{\bar{\rho }}$$

Here, $\bar{\rho }$ is the average dislocation density, and it can be calculated by [25]:

(8) $$\bar{\rho }=\frac{1}{n}\sum_{i=1}^{n}\rho_{i}$$

where n is the total number of cells in the established two-dimensional CA model.

### 3.3. Model of DRX Nucleation

Before reaching the peak stress, unbalanced competition between work hardening and dynamic recovery leads to an increase in dislocation density. When the dislocation density reaches a certain critical value, the nucleation of DRX on the pre-existing grain boundary will be induced. In comprehensive consideration of free energy changes, Roberts and Ahlblom proposed a critical dislocation density, given by [26,27]:

(9) $$\rho_{c}={\left[\frac{20\gamma_{i}\dot{\varepsilon }}{3blM\tau^{2}}\right]}^{1/3}$$

where $\rho_{c}$ is the critical dislocation density; $\gamma_{i}$ denotes the grain boundary energy; $\dot{\varepsilon }$ is the strain rate; l is the mean free path of the dislocation; M and τ are grain boundary mobility and the dislocation line energy, respectively. *l* can be calculated by [28]:

(10) l=KGb/σ

where K represents a constant, approximately equal to 10 for most metal materials; σ is the flow stress. In addition, M and τ can be expressed using the following equations:

(11) $$\tau ={\mathrm{aGb}}^{2}$$

(12) $$M=\frac{\delta D_{\mathrm{ob}}b}{k_{B}T}\exp\left[-\frac{Q_{b}}{\mathrm{RT}}\right]$$

In which a is usually a constant; δ is the characteristic grain boundary thickness; $D_{\mathrm{ob}}$ is the boundary self-diffusion coefficient; $Q_{b}$ is the boundary diffusion activation energy, and $k_{B}$ is Boltzmann’s constant; R is the ideal gas constant.

Many different models have been established to describe the nucleation process of DRX. For example, Ding and Guo [29] proposed that the nucleation rate for DRX behavior is a function of the deformation temperature and the strain rate, described as Equation (13):

(13) $$\dot{n}(\dot{\varepsilon },T)=C_{d}{\dot{\varepsilon }}^{m}\exp\left[-\frac{Q_{\mathrm{act}}}{\mathrm{RT}}\right]$$

where $C_{d}$ is a constant; m is the strain rate sensitivity index; $Q_{\mathrm{act}}$ represents hot deformation activation energy.

### 3.4. Model of DRX Grain Growth

The growth of *R*-grains is essentially the migration of high-angle grain boundaries. Since the dislocation density inside the newly formed *R*-grains is extremely low and approximately equal to zero, a larger dislocation density difference will be generated between the *R*-grains and the strained original grains in the matrix. The stored energy provides a driving force for grain boundary migration through its own reduction, and the changed energy can be composed of two parts, namely the volume energy and the surface energy. Assuming spherical grains, the changed energy can be defined as follows:

(14) $$\mathrm{dE}={\mathrm{dE}}_{\mathrm{vol}}+{\mathrm{dE}}_{\mathrm{surf}}=\frac{4}{3}{\pi r}_{i}{}^{3}\tau (\rho_{i}-\rho_{m})+4\pi r_{i}{}^{2}\gamma_{i}$$

(15) $$r_{i}=\sqrt{\frac{N_{i}A_{c}}{\pi }}$$

where ${\mathrm{dE}}_{\mathrm{vol}}$ is the changed volume energy; ${\mathrm{dE}}_{\mathrm{surf}}$ is the changed surface energy; $\rho_{i}$ is the dislocation density of ith *R*-grain; $\rho_{m}$ is the dislocation density of matrix phase; $r_{i}$ is the radius of ith *R*-grain; $N_{i}$ is the cells number of the ith *R*-grain; $A_{c}$ is the area of one cell. Then, the total driving force F for grain boundary migration can be determined as:

(16) $$F=-\frac{\mathrm{dE}}{\mathrm{dr}}=4\pi r^{2}\tau \Delta \rho -8\pi r\gamma$$

The migration rate of grain boundary $\nu_{i}$ can be expressed as [30]:

(17) $$\nu_{i}=M\Delta f_{i}$$

(18) $$\Delta f_{i}=-F/4\pi r_{i}^{2}=\tau \Delta \rho -2\frac{\gamma_{i}}{r_{i}}$$

where $\Delta f_{i}$ represents the driving force per unit area; $\gamma_{i}$ is the grain boundary energy and can be calculated based on the Read-Shockley equation, as follows:

(19) $$\gamma_{i}=\{\begin{array}{cc} \gamma_{m} & \theta_{i}\geq 15^{{}^{\circ}}\\ \gamma_{i}=\gamma_{m}\frac{\theta_{i}}{\theta_{m}}\left[1-\ln\left(\frac{\theta_{i}}{\theta_{m}}\right)\right] & \theta_{i}\leq 15^{{}^{\circ}} \end{array}$$

where $\gamma_{i}$ is the grain boundary energy; $\theta_{i}$ is the misorientation between the ith crystal grain and its neighboring grains; and $\theta_{m}$ and $\gamma_{m}$ represent the orientation difference and grain boundary energy at high angle boundary (usually taken as 15°), respectively, and $\gamma_{m}$ can be determined as [23]:

(20) $$\gamma_{m}=\frac{{\mathrm{Gb}\theta }_{m}}{4\pi (1-\mu )}$$

where μ represents the Poisson’s ratio. In this study, relevant parameters in the CA model can be obtained by thermodynamic software JMatPro and hot compression test, which are listed in Table 2.

### 3.5. Conditions and Parameters of the Model

**Figure 4:**
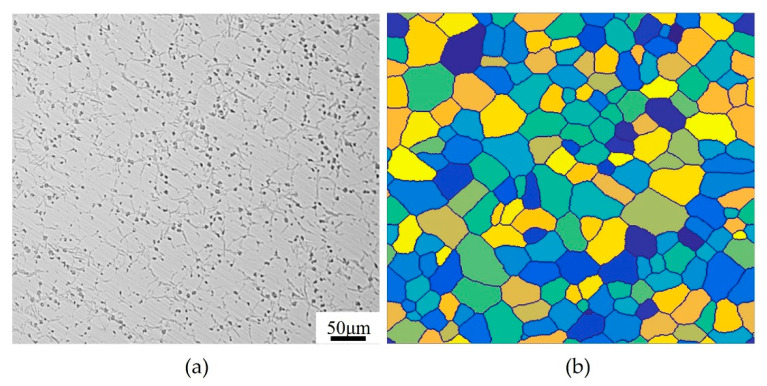
The initial microstructure of (**a**) the actual sample and (**b**) the CA model.

Figure 4 shows the initial microstructure of the actual sample and the CA model. In this study, the initial microstructure needs to be acquired first, which is a prerequisite for numerical simulation work. The initial microstructure is mainly composed of equiaxed grains with an average size of about 40 μm measured by ImageJ software, as shown in Figure 4a. On this basis, a two-dimensional CA model with a size of 500 × 500 square lattices was established to simulate the microstructural evolution of T15MN HSS during DRX. The average grain size $D_{0}$ in CA model was determined by the method of equivalent circle diameter, which can be calculated by:

(21) $$D_{0}={\left(4n_{c}a_{c}^{2}/\pi \right)}^{1/2}$$

where $n_{c}$ is the cell number of a grain; $a_{c}$ is the cell size, set as 1.0 μm, and thus the simulation range in the CA model corresponds to 500 μm × 500 μm in the real sample. In addition, the periodic boundary condition and the Moore neighborhood type are adopted in the simulated mesh model. Before simulating deformation, crystal nuclei with different orientations are firstly randomly dispersed in the cell space, and the initial microstructure with an average of approximately 39.5 μm is obtained through equiprobability growth and curvature-driven growth, as shown in Figure 4b.

During simulation, there are four state variables for each cell in the CA model, which are the grain orientation variable; dislocation density variable; grain boundary recognition variable and recrystallization recognition variable, respectively. The grain orientation variable is used to distinguish different grains and grain boundary energy. Each cell is randomly assigned an orientation angle within the range of 0–180°, and the adjacent cells with the same orientation angle are classified as one grain; the dislocation density variable may record the varication of dislocation density in grains with strain and the deformation stored energy; the grain boundary variable can recognize whether cells are located at grain boundaries; the recrystallization variable can recognize the formation and proportion of *R*-grains. In addition, the influence of carbides on the CA model is not considered in this study.

During the simulation process, three assumptions are made as follows [31]:

(1) Before the deformation of the samples, the initial dislocation density $\rho_{\mathrm{in}}$ of each original grain within the matrix is equal and evenly distributed. The dislocation density will increase as the strain increases, and when reaching the critical dislocation density $\rho_{c}$, DRX is induced.
(2) The initial dislocation density of the newly formed *R*-grains is assumed to be zero, and its value also increases with the continuous increase of strain.
(3) The nucleation of DRX occurs only at the original grain boundaries and the *R*-grains boundaries.

**Figure 5:**
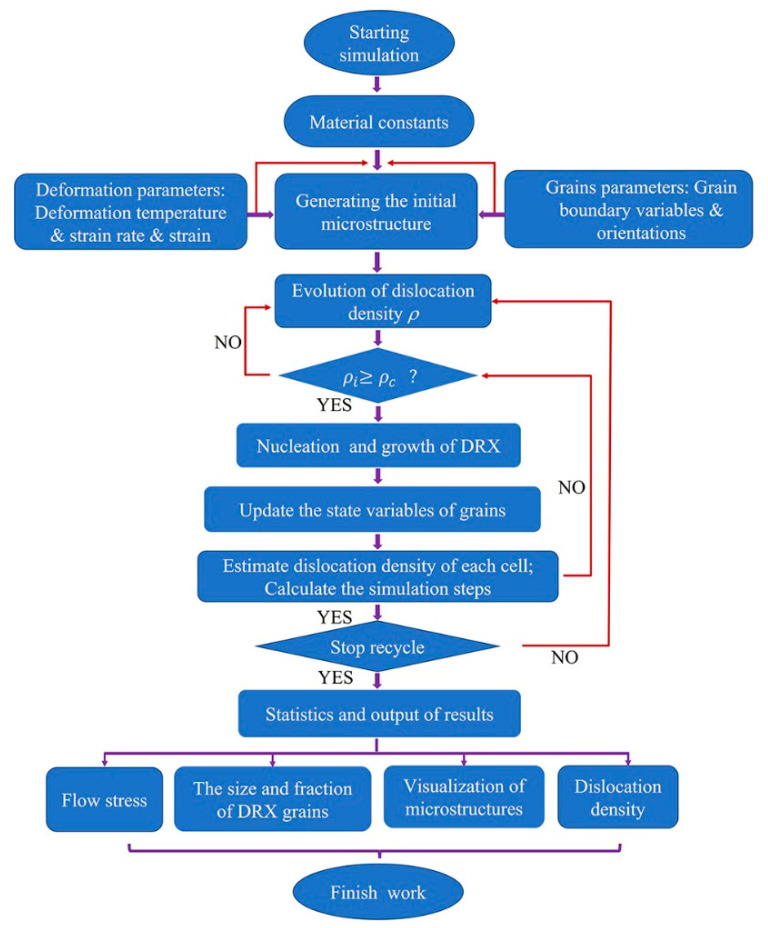
Flowchart of DRX simulation via CA method.

Additionally, Figure 5 depicts the flowchart of DRX simulation for T15MN HSS based on a CA model in this study.

## 4. Results and Discussion

### 4.1. True Stress–Strain Curves

**Figure 6:**
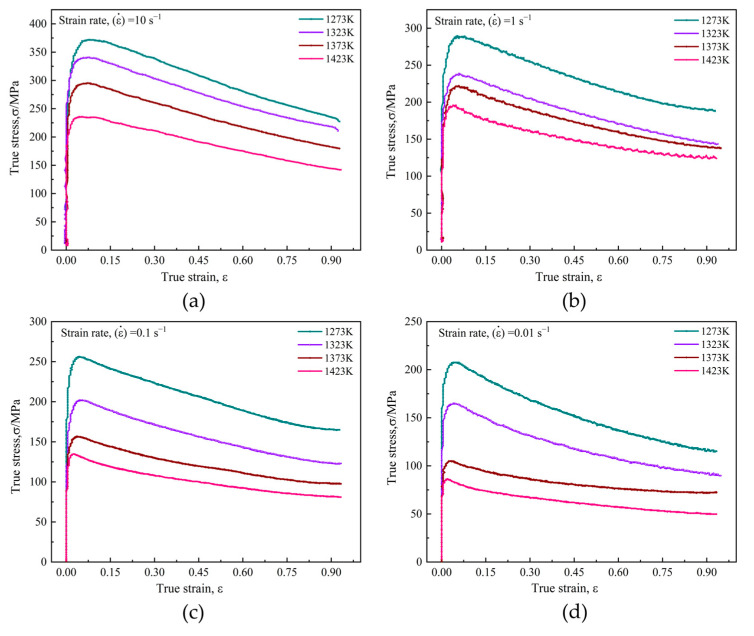
Flow stress–strain curves of T15MN HSS undergoing compression: (**a**) $\dot{\varepsilon }$ = 10 s^−1^; (**b**) $\dot{\varepsilon }$ = 1.0 s^−1^; (**c**) $\dot{\varepsilon }$ = 0.1 s^−1^; (**d**) $\dot{\varepsilon }$ = 0.01 s^−1^.

The true stress–strain (σ–ε) curves of T15MN HSS were obtained based on the hot compression tests at different deformation conditions, as depicted in Figure 6. These curves show that the flow stress varies with various deformation conditions, such as deformation temperature, strain rate and deformation degree (viz., strain). In addition, obviously, all the curves have a similar variation tendency and can be characterized as a single peak, representing the occurrence of DRX for the samples [32]. At the initial stage of compression, the flow stress value increases monotonously with increasing strain due to the work hardening effect caused by the formation and accumulation of numerous dislocations. Meanwhile, as a softening mechanism, DRV competes with work hardening by means of cross slip, climb or rearrangement of dislocations, etc. Subsequently, when the dislocation increases to a critical value ($\rho_{c}$), because of the ongoing plastic deformation, the nucleation of DRX is also stimulated. Before reaching the peak stress, however, dynamic softening (DRV and DRX) is insufficient to completely counteract the effect of work hardening [33]. When the dynamic softening rate is equivalent to the work hardening rate, the flow peak stress is reached. After exceeding the peak stress, the effect of DRX continues to increase and dynamic softening dominates instead of work hardening, causing a gradual decrease in flow stress value until a steady stage, unchanged with strain rising.

Given a strain rate, the flow stress gradually decreases markedly as deformation temperature rises. In addition, especially for lower strain rates, the effect of temperature on rheological behavior seems to be more notable, as shown in Figure 6c,d. By comparison, as for a fixed deformation temperature, the flow stress increases with the increase of strain rate. In other words, flow stress will decrease with decreasing strain rate and increasing deformation temperature. The reason for this phenomenon may be explained as follows [34,35,36]: the reduction in strain rate can guarantee a longer period for the nucleation and growth of DRX, thus weakening the effect of the work hardening at a lower strain rate. and meanwhile, a higher deformation temperature will provide more thermal energy for dislocation motion and promote the mobility of grain boundaries and *R*-grains growth, thereby reducing the work hardening effect. Therefore, it can be concluded that both low strain rate and high temperature are beneficial in reducing the flow stress level.

### 4.2. Constitutive Equations and Hot Deformation Activation Energy

The hot deformation process of metal materials is dependent on thermodynamic parameters, and thus is regarded as a thermally activated process [37]. The hyperbolic-sine type equation including deformation activation energy, first proposed and validated by Sellars and Mctegart [38,39], is widely employed to describe the relationship among flow stress (σ), temperature (T) and strain rate ($\dot{\varepsilon }$), etc., especially at elevated temperature, expressed as:

(22) $$\dot{\varepsilon }=A{\left[\sinh\left(\alpha \sigma \right)\right]}^{n}\exp\left(-\frac{Q_{\mathrm{act}}}{\mathrm{RT}}\right)$$

where A, α are constants independent of experimental conditions; σ is true stress; n is the stress exponent; $Q_{\mathrm{act}}$ is the activation energy of hot deformation; R is the gas constant and T is the chosen deformation temperature.

Other forms of expressions are also applied for depicting the relationship between flow stress and deformation parameters, such as a power-law form (Equation (23)), mainly used to describe low stress (ασ < 0.8), whereas an exponential form (Equation (24)) is preferred for relatively high stress (ασ > 1.2), given by [36]:

(23) $${\dot{\varepsilon }=A}_{1}\sigma^{n_{1}}$$

(24) $$\dot{\varepsilon }=A_{2}\exp\left(\beta \sigma \right)$$

where $A_{1},\;A_{2},\;n_{1},\;\beta$ are material constants and $\alpha =\beta /n_{1}$.

To calculate the parameter values of the constitutive equation, take the natural logarithm of the two sides of Equations (22)–(24) and rearrange the following formulas:

(25) $$\ln\dot{\varepsilon }=\ln A_{1}+n_{1}\ln\sigma$$

(26) $$\ln\dot{\varepsilon }=\ln A_{2}+\beta \sigma$$

(27) $$\ln\dot{\varepsilon }=\mathrm{lnA}+\mathrm{nln}\left[\sinh\left(\alpha \sigma \right)\right]-\frac{Q_{\mathrm{act}}}{\mathrm{RT}}$$

According to Equations (25)–(27), the values of β, $n_{1}$ and n can be determined by solving the slope average values of the plots of $\ln\dot{\varepsilon }\mathrm{vs}.\;\sigma$, $\ln\dot{\varepsilon }\mathrm{vs}.\;\ln\sigma$, as well as $\ln\dot{\varepsilon }\mathrm{vs}.\;\ln[\sinh(\alpha \sigma )]$, respectively. The applied equations are as follows:

(28) $$\beta ={\left[\frac{\partial \ln\dot{\varepsilon }}{\partial \sigma }\right]}_{T}$$

(29) $$n_{1}={\left[\frac{\partial \ln\dot{\varepsilon }}{\partial \ln\sigma }\right]}_{T}$$

(30) $$n={\left[\frac{\partial \ln\dot{\varepsilon }}{\partial \ln\left[\sinh\left(\alpha \sigma \right)\right]}\right]}_{T}$$

Here, it is assumed that the activation energy of hot deformation ($Q_{\mathrm{act}}$) is independent of temperature and strain rate. Taking the partial derivative of both sides of Equation (27), the following equation can be derived:

(31) $$Q_{\mathrm{act}}=R{\left[\frac{\partial \ln\dot{\varepsilon }}{\partial \ln\left[\sinh\left(\alpha \sigma \right)\right]}\right]}_{T}{\left[\frac{\partial \ln\left[\sinh\left(\alpha \sigma \right)\right]}{\partial \left(1/T\right)}\right]}_{\dot{\varepsilon }}=\mathrm{Rn}K$$

**Figure 7:**
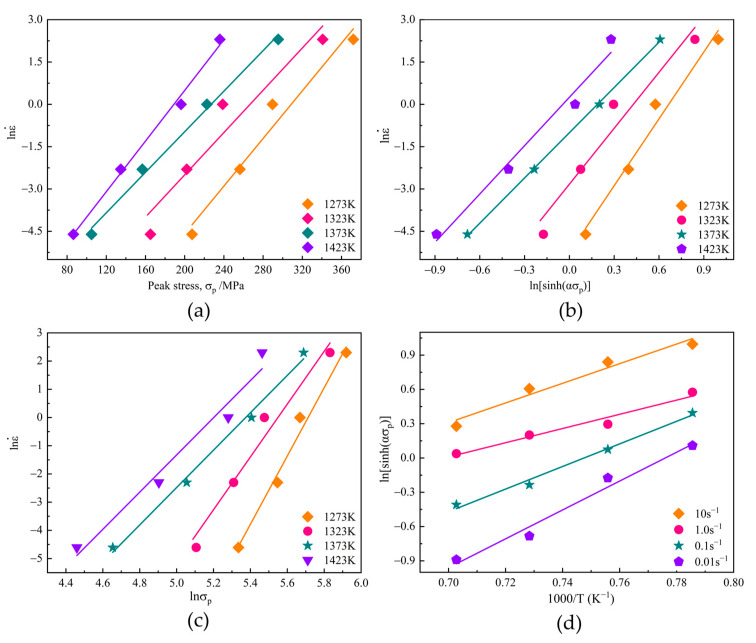
All of the plots used for determining the parameters of the constitutive equation: (**a**) $\ln\dot{\varepsilon }\mathrm{vs}.\;\sigma_{p}$; (**b**) $\ln\dot{\varepsilon }\mathrm{vs}.\;\ln\sigma_{p}$; (**c**) $\ln\dot{\varepsilon }\mathrm{vs}.\;\ln[\sinh(\alpha \sigma_{p})]$; (**d**) $\ln\left[\sin h\left({\alpha \sigma }_{p}\right)\right]\;\mathrm{vs}.\;1000/T$.

By consideration of the peak stress ($\sigma_{p}$) under different deformation conditions, the required plots of $\ln\dot{\varepsilon }\mathrm{vs}.\;\sigma_{p}$ and $\ln\dot{\varepsilon }\mathrm{vs}.\;\ln\sigma_{p}$ are depicted in Figure 7a,b. The mean values of the line slopes are determined by linear regression, and β and $n_{1}$ are calculated as 0.0402 and 8.707, respectively, and so the value of α can be obtained, viz., $\alpha =\beta /n_{1}$ = 0.00462. Likewise, the relationship curves of $\ln\dot{\varepsilon }\mathrm{vs}.\;\ln[\sinh(\alpha \sigma_{p})]$, and $\ln\left[\sin h\left({\alpha \sigma }_{p}\right)\right]\;\mathrm{vs}.\;1000/T$ are also plotted. The average values of n and *K* are calculated as 6.413 and 9.35, respectively. Consequently, $Q_{\mathrm{act}}=\mathrm{Rn}K=$ 498.520 kJ/mol.

Additionally, the Zener-Hollomon parameter (Z parameter) is usually utilized to characterize the effects of temperature and strain rate on flow stress behavior, which is defined by:

(32) $$Z=\dot{\varepsilon }\exp\left(\frac{Q_{\mathrm{act}}}{\mathrm{RT}}\right)=A{\left[\sinh\left(\alpha \sigma \right)\right]}^{n}$$

Therefore, the constitutive equation containing the Z parameter can be expressed as follows:

(33) $$\sigma =\frac{1}{\alpha }\ln\left\{{\left(\frac{Z}{A}\right)}^{1/n}+{\left[{\left(\frac{Z}{A}\right)}^{2/n}+1\right]}^{1/2}\right\}$$

**Figure 8:**
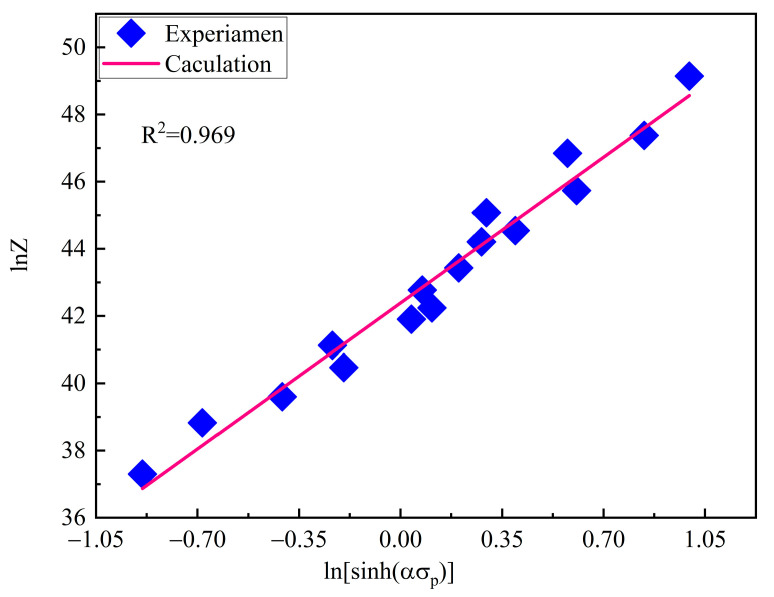
Relationship between the Z parameter and peak stress.

Figure 8 shows the plots of $\mathrm{lnZ}\;\mathrm{vs}.\;\ln\left[\sin h\left({\alpha \sigma }_{p}\right)\right]$ obtained by linear regression method. The value of lnA is determined as 42.388 by finding the intercept of the fitted line, thus obtaining A = 2.564 × 1018. It can be evidently seen that the Z parameter increases monotonically with increasing peak stress, and the linear correlation coefficient of the fitting line is 0.969, which partly elucidates that the flow stress behavior of T15MN HSS can be well expressed by the Z parameter hyperbolic sine function during hot deformation.

Based on the above expressions and the solved parameters, the dynamic equation of T15MN HSS at elevated temperature during hot deformation can be obtained, as shown below:

(34) $$\dot{\varepsilon }=2.564\times 10^{18}{\left[\sinh\left(0.00462\sigma \right)\right]}^{6.413}\exp\left(-\frac{498,520}{8.314T}\right)$$

Additionally, the constitutive equations with the Z parameter are as follows:

(35) $$\sigma =\frac{1}{0.00462}\ln\left\{{\left(\frac{Z}{2.564\times 10^{18}}\right)}^{1/6.413}+{\left[{\left(\frac{Z}{2.564\times 10^{18}}\right)}^{2/6.413}+1\right]}^{1/2}\right\}$$

(36) $$Z=\dot{\varepsilon }\exp\left(\frac{498,520}{8.314T}\right)$$

### 4.3. Simulation of DRX Process

#### 4.3.1. The Evolution of DRX Microstructure

**Figure 9:**
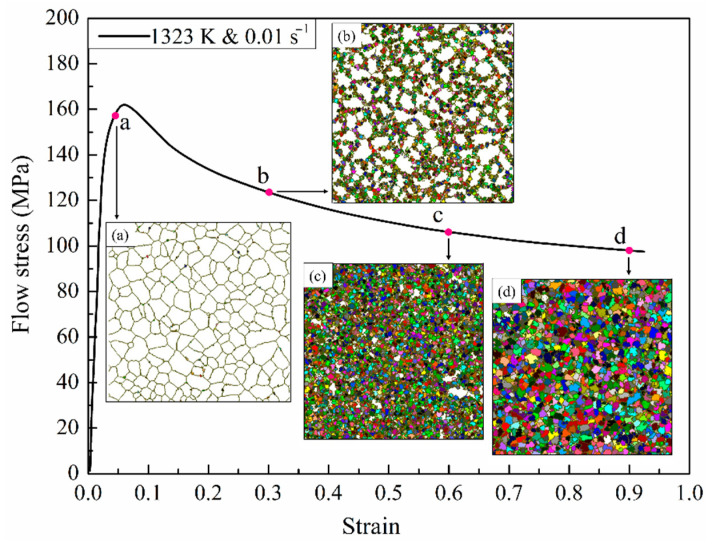
The flow stress curve and corresponding to the microstructural evolution of T15MN HSS with strain by CA method at 1323 K and 0.01 s^−^^1^: (**a**) ε = 0.05; (**b**) ε = 0.3; (**c**) ε = 0.6; (**d**) ε = 0.9.

The visual prediction of experimental results is very meaningful in further revealing the mechanism of DRX. Based on a mesoscopic CA model, the dynamic evolution process of the deformed microstructure of the samples under compression can be monitored more intuitively through the graphical form, which may be divided into three main procedures: the nucleation, spread and growth of recrystallization grains [40]. Figure 9 illustrates the simulated flow stress–strain curve and the microstructural evolution at a temperature of 1323 K and strain rate of 0.01 s^−1^ during hot deformation. The white regions and colorful regions represent original grain and *R*-grains in a deformed matrix, respectively. Following the pre-set nucleation principle, the *R*-grains are mainly formed along the grain boundaries. The simulation results make it clear that the fraction and the average size of *R*-grains generally increase with the increase of strain. Furthermore, it is noticed that when the strain is 0.05 (corresponding to Point a), a sporadic newly formed crystal nucleus has already appeared at the grain boundaries, implying that the strain in the local region reaches a higher strain value than the critical strain $\varepsilon_{c}$. Therefore, the increasing rate of flow stress is visibly slowed down. When the strain is 0.3 (corresponding to Point b), plenty of new grains nucleate and gradually extend into the grains with increasing strain, and the average size of the DRX grains increases as well. At this moment, the dynamic softening dominated by XRD gradually counteracts and exceeds the effect of work hardening, causing a decrease in flow stress. When the strain reaches 0.6 (corresponding to Point c), the *R*-grains dominate compared with the original grains. For a strain value of 0.9 (corresponding to Point d), the complete DRX process is basically achieved, and the average size of *R*-grains increases appreciably.

#### 4.3.2. Prediction of Dislocation Density

**Figure 10:**
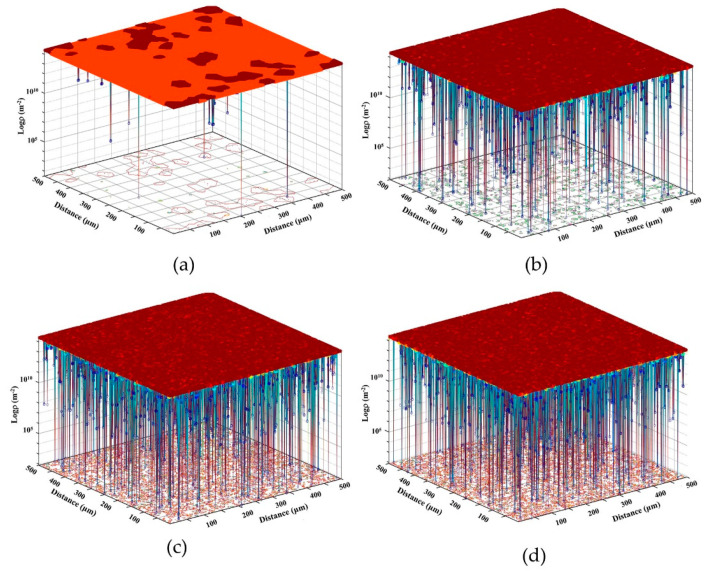
Dislocation density distribution of T15MN HSS during DRX at 1323 K and 0.01 s^−1^ via CA method with various strains of (**a**) 0.05; (**b**) 0.3; (**c**) 0.6; (**d**) 0.9.

Dislocation density is a crucial criterion in terms of determining whether the DRX process occurs, so it is an important factor usually involved in the study of the thermal deformation of metal materials. The microstructural evolution of materials is dependent on the variation of dislocation density inside deformed grains, and because only when the dislocation density reaches and exceeds the critical dislocation density $\rho_{c}$, DRX behavior will occur. To better understand the mechanism of the DRX process, the evolution of dislocation density corresponding to the microstructural evolution described above needs to be analyzed. Figure 10 illustrates the distribution maps of dislocation density evolution with the strains of 0.05, 0.3, 0.6, 0.9, respectively, by utilizing a 2D DRX-CA model at the temperature of 1323 K and a strain rate of 0.01 s^−1^, where the *z*-axis is represented by the logarithm of the dislocation density.

It can be found from Figure 10 that the average dislocation density at the initial stage of hot compression deformation is low, and then increases rapidly with increasing strain due to the formation and accumulation of new dislocations. At the initial stage, *R*-grains begin to nucleate at local grain boundaries. However, the mean dislocation density at this time is still very low, because the dislocation density *ρ* reaches and exceeds the critical value $\rho_{c}$ only in a few scattered local regions of grain boundaries in the initial microstructure, just as shown in the microstructure of Figure 9a (corresponding to Point a). With the progression of plastic deformation, the average dislocation density also increases gradually (see Figure 10b), and there will be more regions along the grain boundary satisfying the conditions of nucleation, corresponding to Point b on the stress–strain curve in Figure 9. There is a dislocation density difference between the *R*-grains and the original grains in the matrix, which provides a driving force for the growth of the *R*-grains and consequently promotes the gradual growth of the grains. While at the subsequent stage of thermoplastic deformation, the average dislocation density varies little and approximates to a stable state of nucleating easily, attributing to the joint action of multiple mechanisms, as shown in Figure 10c,d.

#### 4.3.3. Effect of Deformation Temperature and Strain Rate on Microstructure

**Figure 11:**
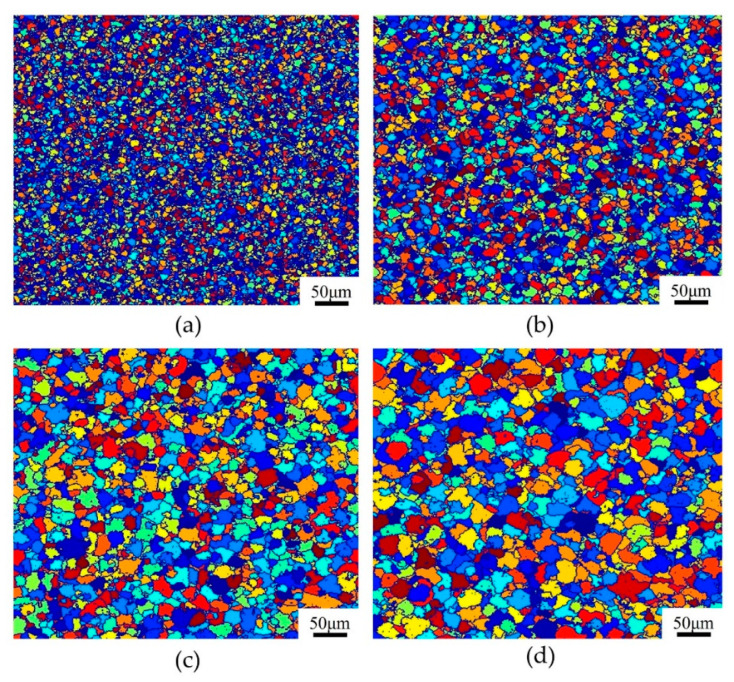
Simulated microstructural evolution of T15MN HSS with the strain of 0.9 at a strain rate of 0.1 s^−1^ and deformation temperatures of (**a**) 1273 K; (**b**) 1323 K; (**c**) 1373 K; (**d**) 1423 K.

**Figure 12:**
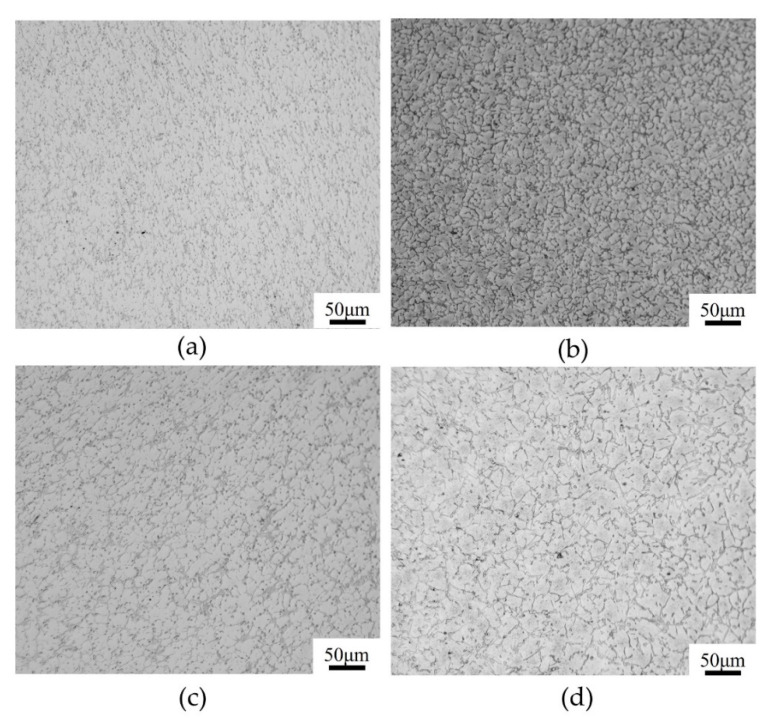
Metallographic structure of T15MN HSS undergoing compression with the strain of 0.9 at a strain rate of 0.1 s^−1^ and different deformation temperatures of (**a**) 1273 K; (**b**) 1323 K; (**c**) 1373 K; (**d**) 1423 K.

The simulated DRX microstructures of T15MN HSS at a strain rate of 1.0 s^−1^ and the deformation temperature range of 1273–1423 K, with a true strain of 0.9, were captured using the CA method, as presented in Figure 11. The results show that the effect of deformation temperature on the recrystallized microstructure is glaringly obvious. In addition, when the strain rate is set to a constant, the average size of dynamic *R*-grains increases gradually as the deformation temperature rises. The growth of *R*-grains is attributed to the high atomic diffusion rate and the mobility of the grain boundaries at elevated temperatures [30]. Additionally, increasing the deformation temperature possesses the capability of shortening the incubation period of DRX and promoting rapid nucleation of *R*-grains at the particles or defects of the grain boundaries. Of course, too high a temperature also leads to the coarsening of *R*-grains. For example, when the deformation temperature is increased from 1273 K to 1423 K at an interval of 50 K, the corresponding values of the average *R*-grain size based on CA simulation are 7.16 μm, 11.57 μm, 15.73 μm, and 21.83 μm, respectively. This characteristic is consistent with the corresponding optical microstructures of T15MN HSS under the same conditions (see Figure 12). The corresponding values of the average *R*-grain size obtained by metallographic analysis were determined to be 6.91 μm, 11.12 μm, 15.36 μm, and 20.26 μm respectively. Therefore, the error between the experimental values and the calculated ones is, overall, acceptable.

**Figure 13:**
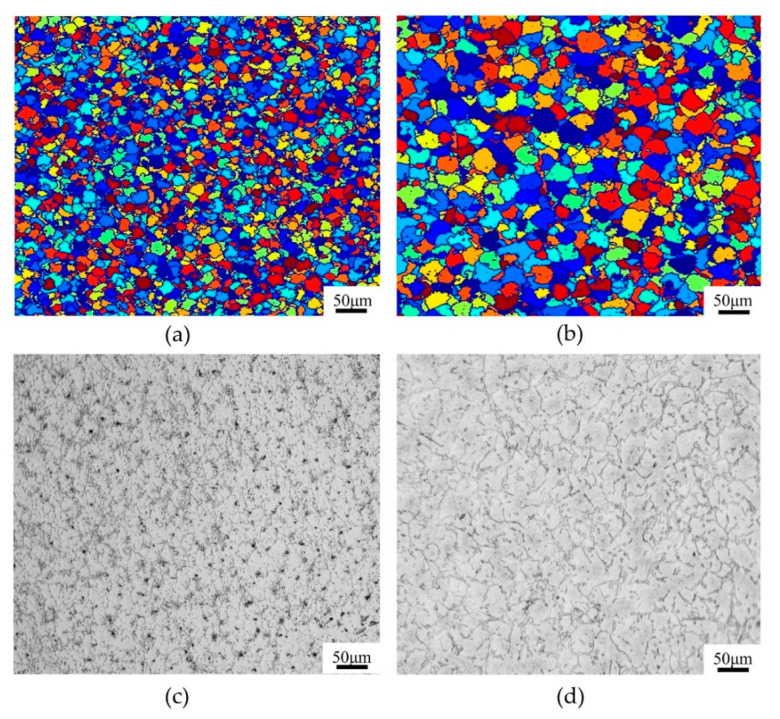
Comparisons of microstructure between simulated results and experimental ones with a true strain of 0.9 at the temperature of 1373 K and different strain rates of (**a**,**c**) 1.0 s^−1^; (**b**,**d**) 0.01 s^−1^.

Under specified conditions, the effect of strain rate on recrystallization behavior was also studied. A series comparison of the DRX microstructure of T15MN HSS at a temperature of 1373 K and strain rates of 0.01 s^−1^ and 1.0 s^−1^ was performed by experimental compression and simulation, corresponding to a true strain of 0.9, and the results of the comparison are displayed in Figure 13. As can be seen from the simulated results, the average size and volume fraction of *R*-grains increase with decreasing strain rate, and the average *R*-grain size by simulation was 14.11 μm and 25.12 μm, respectively (see Figure 13a,b). It is common knowledge that a slower strain rate can provide more time for the recrystallization process, i.e., more sufficient nucleation and growth of *R*-grains. Meanwhile, with increasing strain rate, the relative time before the onset of DRX behavior is extended. At the relatively higher strain rate of 1.0 s^−1^, the recrystallization process may not be fully completed, which prevents the *R*-grains from growing sufficiently during the growth cycle, owing to the lack of sufficient time, while the dislocation density also increases. It can be concluded that a slower strain rate is favorable for the nucleation and growth of DRX grains, but may cause the coarsening of *R*-grains. Meanwhile, a higher strain rate can generate fine recrystallization grains, but may cause recrystallization to not occur sufficiently. The simulation results are further confirmed by the experimental ones, the average *R*-grain size of which were measured as 13.27 μm and 24.77 μm, respectively, as illustrated in Figure 13c,d.

The object of thermal deformation (viz., hot working) is to eliminate microstructure defects and refine grains. As noted above, a higher temperature or lower strain rate causes grain coarsening, while a lower temperature or higher strain rate may lead to an inadequate recrystallization process. However, a uniform microstructure and appropriate grain size are desirable. As far as the above image results are concerned, the microstructure obtained under the deformation conditions of 1373 K and 1.0 s^−1^ or 1323 K and 0.1 s^−1^ seem more appropriate for subsequent heat treatment processes, including quenching and tempering.

#### 4.3.4. Verification of Flow Stress Curves

**Figure 14:**
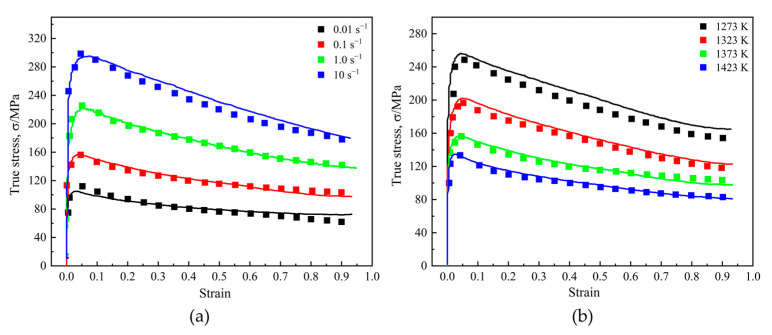
A comparison of flow stress curves of T15MN HSS between the experiment values and predicted ones at (**a**) 0.1 s^−1^ and 1273–1423 K; (**b**) 1373 K and 0.01–10 s^−1^.

To further validate the accuracy of the proposed DRX-CA model, simulation work was also carried out on the flow stress behavior of the deformed material. The prediction values of the flow stress obtained by CA simulation at 1273–1423 K and 0.1 s^−1^ and 0.01–10 s^−1^ and 1373 K are depicted and compared with the corresponding experimental results, as shown in Figure 14. Overall, the simulation results indicate that the prediction values are in good agreement with the experimental ones. It can be seen from the phase diagram in Figure 2, above, within the set temperature range of 1273–1423 K, that the matrix microstructure of the sample before compression testing is mainly composed of austenite and partly of carbides. Consequently, the discrepancy between the prediction values and the experimental ones may be affected by the existential state of the carbides, such as their quantity, shape, size, and distribution. Additionally, microstructure defects and other two-phase particles of the actual material can also be the cause of errors, as well as the process assumptions, transformation rules, and model parameters, especially the internal dislocation density, which is difficult to measure, and which seems to indicate the established DRX-CA model still needs to be improved in terms of transformation rules, parameters, or other factors for suiting different cases.

## 5. Conclusions

The hot deformation behavior of T15MN HSS during isothermal compression was evaluated through the method of combining experiments with CA simulation. Some of the main conclusions of this research can be summarized as follows:

(1) All the flow stress curves are characterized by a single peak, which indicates the occurrence of DRX behavior. Additionally, flow stress is very sensitive to deformation temperatures, strain rates, and strain. True stress will decrease with decreasing strain rate and increasing deformation temperature. Based on experimental analysis, the constitutive equation and thermal activation energy are obtained. In this regard, the Arrhenius equations containing the Z parameter can be expressed as:
  (37) $$\sigma =\frac{1}{0.00462}\ln\left\{{\left(\frac{Z}{2.564\times 10^{18}}\right)}^{1/6.413}+{\left[{\left(\frac{Z}{2.564\times 10^{18}}\right)}^{2/6.413}+1\right]}^{1/2}\right\}$$
  (38) $$Z=\dot{\varepsilon }\exp\left(\frac{498,520}{8.314T}\right)$$
(2) The concepts and components of the cellular automata model were introduced, mainly including component cell, cell space, neighbor types, and transformation rules. In addition, the models of dislocation density evolution, nucleation, and growth of DRX grains were illustrated and integrated into the model. Then, a 2D CA model was established in this study to describe the DRX behavior of T15 HSS, which was implemented on the MATLAB platform. This method provided a mesoscopic model to bridge the macroscopic hot-working process with the microscopic DRX behavior, enabling real-time visualization of the microstructural evolution of metal materials during hot deformation.
(3) Based on the proposed DRX-CA model, an investigation was performed on the effect of deformation parameters on the evolution and prediction of microstructure, variation of dislocation density, and flow stress behavior. Both the increased deformation temperature and the decreased strain rate can promote an increase in the average size and fraction of *R*-grains, as well as the dislocation density. The good agreement between the experimental and simulation results indicates that the established DRX-CA model can provide a certain theoretical reference for the prediction of microstructure and regulation of properties for the material.

## Figures and Tables

**Table 1:** Chemical composition of T15MN HSS (wt.%).

Element	C	W	Mo	Cr	V	Nb	Co
Content	1.4	12.0	3.0	4.1	2.5	1.0	5.0

**Table 2:** Materials parameters of the studied alloy steel for CA model.

Nomenclature	Physical Meaning	Value	Unit
h	The interaction coefficient of dislocation density	0.5	-
T	Deformation temperature	1273, 1323, 1372, 1423	K
$T_{m}$	Melting point of material	1518	K
$G_{0}$	Shear modulus (303 K)	82.46	GPa
a	Constant	0.5	-
R	Ideal gas constant	8.314	J·mol^−1^·K^−1^
$K_{1}$	Constant	10	-
b	Burger’s vector	0.26	nm
μ	Poisson ratio	0.35	-
$\theta_{m}$	Critical orientation	15	°
$Q_{\mathrm{act}}$	Hot deformation activation energy	498,520	J·mol^−1^
$Q_{b}$	Boundary diffusion activation energy	375,000	J·mol^−1^
$\delta D_{b}$	Boundary self-diffusion coefficient	5 × 10^−15^	m^3^·s^−1^
$k_{B}$	Boltzmann constant	1.38 × 10^−23^	J·K^−1^
$C_{d}$	Material parameter	1	-
m	Strain-rate sensitivity index	1	-

## Data Availability

Data sharing is not applicable to this article.
